# Gut Microbiota as a Missing Link Between Nutrients and Traits of Human

**DOI:** 10.3389/fmicb.2018.01510

**Published:** 2018-07-06

**Authors:** Hea-Jong Chung, Thi T. B. Nguyen, Hyeon-Jin Kim, Seong-Tshool Hong

**Affiliations:** ^1^Department of Biomedical Sciences, Institute for Medical Science, Chonbuk National University Medical School, Jeonju, South Korea; ^2^JINIS BDRD Institute, JINIS Biopharmaceuticals Co., Wanju, South Korea

**Keywords:** gut microbiota, nutrients, human traits, diet, disease

## Contribution of gut microbiota in determining human diseases

With the development of next generation sequencing (NGS) technology, the role of gut microbiota in human health has been extensively studied. Metagenome sequencing analysis, which is based on NGS, and subsequent statistical analysis showed that the relationship between gut microbiota and humans is not merely commensal but rather a mutualistic relationship (Chen et al., 2013; Jandhyala et al., 2015). Recent advances in the field of gut microbiota are elucidating our understanding of human biology.

The microbiota of the human gut is a massive and complex microbial community consisting of 100 trillion microbes in the intestine. The gut microbiota is essential to the health and well-being of the host (Marchesi et al., 2016). Although interactions between gut microbiota and its host have negative effects in some cases, these interactions positively affect the host in most cases. It is now clear that the gut microbiota contributes significantly to the traits of humans as much as our genes, especially in the case of atherosclerosis, hypertension, obesity, diabetes, metabolic syndrome, inflammatory bowel disease (IBD), gastrointestinal tract malignancies, hepatic encephalopathy, allergies, behavior, intelligence, autism, neurological diseases, and psychological diseases (Chen et al., 2013; Nguyen et al., 2017; Zhang et al., 2017; Table 1). It has also been found that alteration of the composition of the gut microbiota in its host affects the behavior, intelligence, mood, autism, psychology, and migraines of its host through the gut-brain axis (Chen et al., 2013). Thus, the effect of the gut microbiota on human phenotypes has become a booming area of research and presents a new paradigm of opportunities for medical and food applications.

**Table 1:** Effect of the gut microbiota on human diseases.

**Disease/ disorder**	**Implicated microbiota**	**Potential role of the microbiome**
**METABOLIC DISEASES**		
Obesity	Firmicutes/Bacteroidetes ratio Prevotellaceae Eubacterium Faecalibacterium Roseburia	Significant changes in gut microbiota are associated with increased obesity
Type II diabetes	Bacteroidetes/Firmicutes Bacteroides-Prevotella *Eggerthella lenta Clostridia Eubacterium rectale Faecalibacterium prausnitzii Roseburia intestinalis Roseburia inulinivorans*	Shifts in gut microbiota are associated with increases in plasma glucose concentrations
Hypertension	*Prevotella Klebsiella Bifidobacterium Butyrivibrio Coprococcus Faecalibacterium Roseburia*	Gut dysbiosis increases hypertension
**IMMUNE DISEASES**		
IBD	Bacteroidetes *Lachnospiraceae Actinobacteria Proteobacteria Clostridium leptum Clostridium coccoides Faecalibacterium prausnitzii* Firmicutes/Bacteroidetes ratio *Bifidobacteria*	Immune response to the gut microbial community Composition of the gut microbiota contributes to inflammation
Allergies	*Lactobacillus* spp. *Bifidobacterium adolescentis Clostridium difficile*	Early colonization with *Lactobacillus* is associated with decreased allergies Early colonization with more diverse microbiota might prevent allergies
Celiac disease	*Bacteroides vulgatus Escherichia coli Clostridium coccoides*	High diversity in Celiac disease patients vs. control
Type I diabetes	*Bacteroides Streptococci Clostridium cluster IV and XIVa*	Interaction between the gut community and the innate immune system may be a predisposing factor for diabetes
Rheumatoid arthritis	Bifidobacteria *Bacteroides Porphyromonas Prevotella Bacteroides fragilis Eubacterium rectale Clostridium coccoides*	Treg-promoting organisms depleted; overgrowth of bacteria that induce Th17 cell populations, leading to inflammation Intestinal microbes associated with etiology
Atopy and asthma	Bifidobacteria *Bacteroides Staphylococcus* spp. *Streptococcus* spp. Enterobacteria *Clostridium difficile*	Pre- and post-natal microbial exposure appear key to appropriate immune development Mode of delivery and nutrient uptake are important factors for GI community development and protection against subsequent atopic disease development
**AUTISM**		
Autism	*Clostridial species*	Increased bacterial diversity in the feces of autistic children compared to control
**PSYCHOLOGICAL DISEASE**		
Anxiety and depression	*Lactobacillus reuteri Lactobacillus rhamnosus Bifidobacterium infantis*	Decreased anxiety and stress-induced increase of corticosterone

## The gut microbiota of humans fluctuates in response to nutritional uptake rather than remaining stably immutable throughout life

Recent studies have elucidated that the gut microbiota plays essential roles in the health and well-being of its host, and whether the composition of the gut microbiota fluctuates or stays constant throughout the lifetime of its host has become one of the main questions to ponder in the scientific community. The prevailing opinion has been that the gut microbiota develops rapidly right after birth and fluctuates only until it matures, which usually takes ~2 years after birth (Koenig et al., 2011). Once the gut microbiota is established, its composition remains stably immutable throughout life. However, recent evidence shows that this opinion is wrong, and the composition of gut microbiota can fluctuate during the lifetime of its host (Wu et al., 2011; David et al., 2014).

It is observed that the growth of almost all microbial organisms is very sensitive to their ambient nutrients. Additionally, considering the diversity and number of microbes in the gut microbiota, it would be more reasonable to speculate that the composition of the gut microbiota could constantly fluctuate, reflecting the diet of its host. Wu et al. recently showed that the long-term consumption of different diets, such as plant-based diets or animal-based diets, drastically altered the composition of gut microbiota, even at the phylum level in the taxonomic hierarchy (Wu et al., 2011). Vegetarian diets consist of fibers containing resistant starch and non-starch polysaccharides. Interestingly, numerous studies have shown that vegetarian diets increased the abundance of carbohydrate-degrading microbes, such as *Prevotella, Roseburia, Eubacterium rectale*, and *Ruminococcus bromii*, in their gut microbiota (Wu et al., 2011; David et al., 2014). In contrast, western diets high in protein and fat, which promote chylomicron and bile acids, increase the abundance of bile acid-tolerant microbes, such as *Alistipes, Bilophila*, and *Bacteroides*, in their gut microbiota (David et al., 2014). A defined food consumption experiment by David et al. even showed that the composition of gut microbiota is promptly affected by the dietary fluctuations within a day. Even cyclical shifts in daily feeding or fasting affected the increase of specific genera in the gut microbiota (David et al., 2014). These studies clearly show that the composition of the human gut microbiota constantly fluctuates in response to the nutritional composition of the diet rather than remaining stably immutable throughout life.

## Nutrients affect the composition of the gut microbiota, and both modified gut microbiota and nutrients affect human traits together

Nutrients are dietary components that an organism metabolizes for survival and growth. Nutrients are substances that provide energy and/or form a component of body tissues. Higher organisms, such as humans, intake nutrients in their diets to maintain the precisely functioning metabolic machinery affecting the health and well-being of the organism. Because nutrients are essential substances for sustaining life, there is much less genetic variation in the genes involved in processing nutrients compared to other genes in humans (Fraser, 2015). Considering that nutrients absorbed by an organism are precisely processed by the well-orchestrated metabolic machinery in the bodies of organisms, diets have a limited ability in terms of affecting the traits of human. However, epidemiological research has proven that diet significantly affects human traits (Sharief et al., 2011; Boada et al., 2016). The quantity of calories and dietary patterns are key determinants of the anthropometric quantitative traits, which are especially reflected in the positive height trend in the developed countries (Jelenkovic et al., 2016). In the context of the nature of nutrients, an association between anthropometric quantitative traits and nutrients is expected. Interestingly, the effect of nutrients on human traits is not limited to anthropometric quantitative traits. Studies on monozygotic twins show that nutrients strongly affect various metabolic diseases and immune diseases (Rissanen et al., 2002; Spehlmann et al., 2012). Epidemiological research has shown that nutrients are considered a fundamental factor along with genetics in the development and/or prevention of rheumatoid arthritis, multiple sclerosis, asthma, and allergies (Sharief et al., 2011; Thorburn et al., 2014). In addition, numerous studies have proven an association between cancer and nutrients (Boada et al., 2016). Because of the clear association between cancer and nutrients, the World Health Organization (WHO) and the International Agency for Research on Cancer (IARC) even declared that hydrogenated oils, potato chips, processed meats, red meats, farmed salmon, and refined sugar are associated with various cancers. These results definitely indicate that human traits are also strongly shaped by nutrients. In this sense, the opinion of “we are what we eat” by the general public would be scientifically wise. Despite the expectation that nutrients only slightly affect human traits, it is surprising to observe that nutrients seem to affect human traits as much as our own genes, which suggests that there might be a strong link between nutrients and human traits.

**Figure 1:**
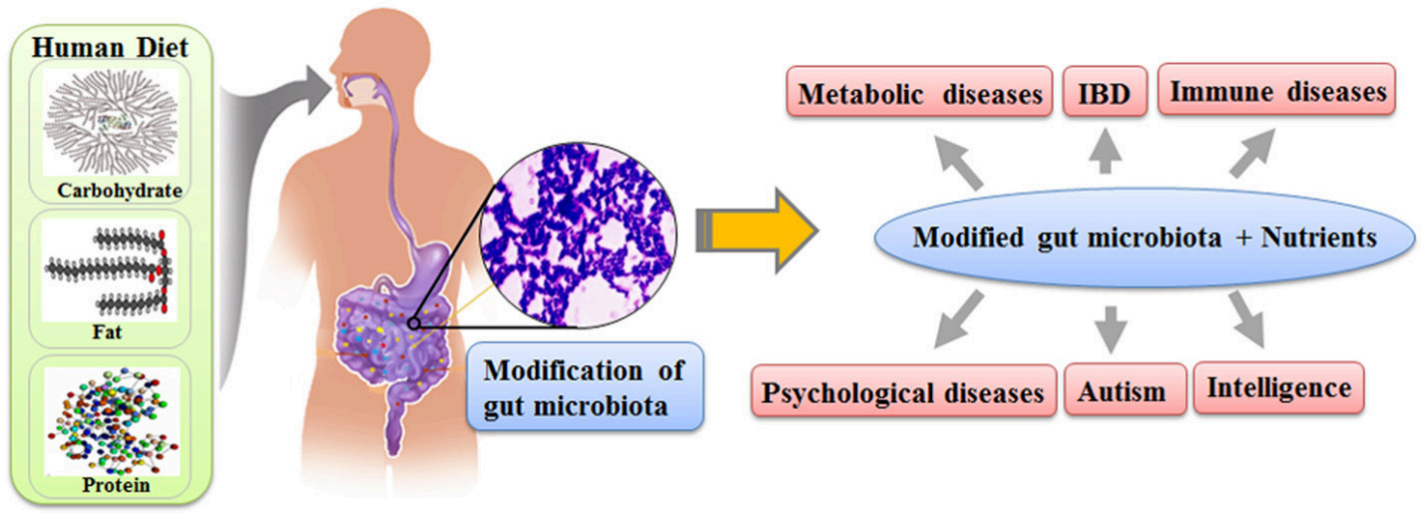
The schematic diagram on how nutrients affect the human traits through gut microbiota.

Recent scientific evidence regarding the gut microbiota makes it possible to explain the link between nutrients and human traits. The gut microbiota not only directly interacts with the somatic cells of its host to affect the traits of human, as in the case of training immunological networks, but also generates various chemicals, which can directly modify the biochemical pathways of humans. The composition of the gut microbiota fluctuates based on the nutrient uptake of its host, and the composition of the gut microbiota affects various human traits as much as our genes (Figure 1). Therefore, it is reasonable to speculate that the effect of nutrients on human traits would be the combined results from both the gut microbiota modified by the nutrient uptake and the nutrients themselves. We believe that the gut microbiota is the missing link between nutrients and modifications of human traits.

## Author contributions

S-TH conceived the idea and designed the structure of the manuscript. H-JC, TN, and S-TH drafted the manuscript, table and figure. All authors have critically read, corrected, and approved the final version of the manuscript and agree with the opinions expressed here.

### Conflict of interest statement

The authors declare that the research was conducted in the absence of any commercial or financial relationships that could be construed as a potential conflict of interest.
